# Supplementary material for: Solution-combustion synthesis of Na3(VO1-x)2(PO4)2F1+2x as a positive electrode material for sodium-ion batteries
Source: Commun Eng. 2025 Aug 5;4:143. doi: 10.1038/s44172-025-00471-w (PMC12325671; doi:10.1038/s44172-025-00471-w)
Supplement: Supplementary file 2 — Supplementary Information [file 44172_2025_471_MOESM2_ESM.pdf]

Supplementary Information

Solution-combustion synthesis of $Na_3(VO_{1-x})_2(PO_4)_2F_{1+2x}$ as a positive electrode material for sodium-ion batteries

Oskar Grabowski, Michal Krajewski, Magdalena Winkowska-Struzik and Andrzej Czerwinski*

Faculty of Chemistry, University of Warsaw, Pasteura 1, 02-093 Warsaw, Poland

E-mail: michal.krajewski@uw.edu.pl

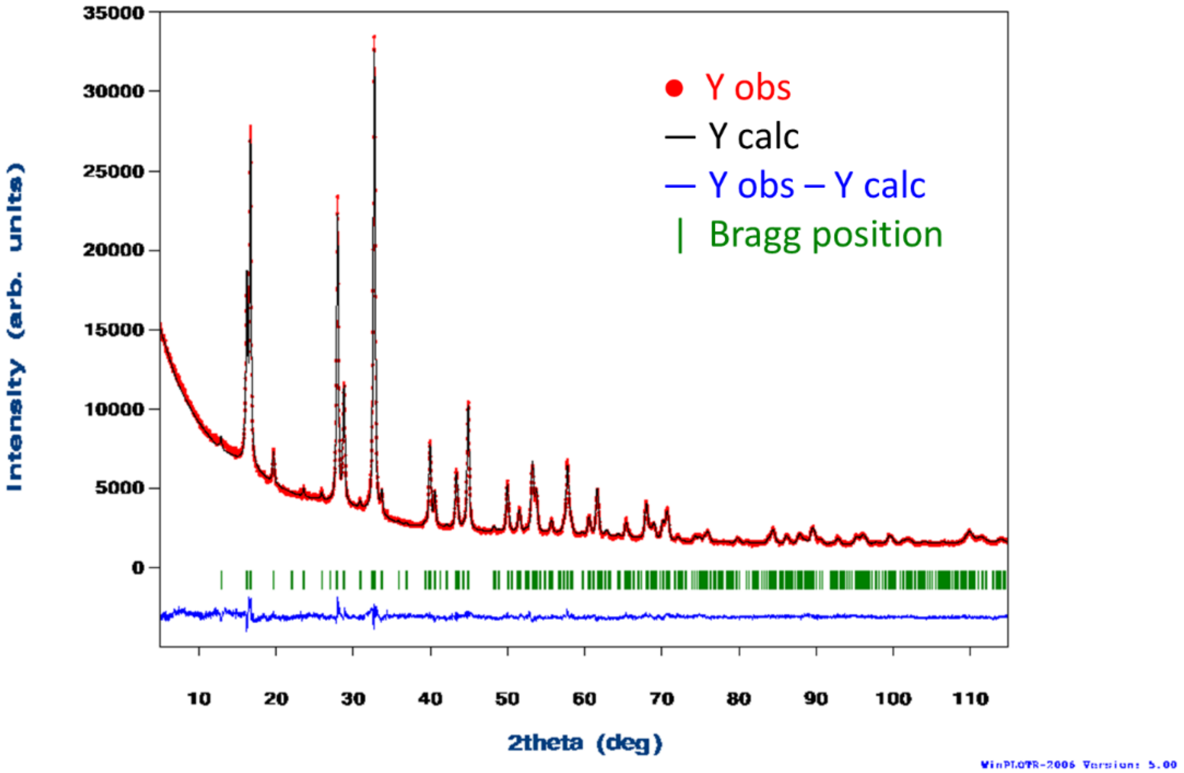

Supplementary Figure 1. Rietveld refinement of powder XRD data collected for NVPF@C sample. The pattern showed good agreement with the P42/mnm space group.

Unit cell (Å)			Volume ($Å^3$)	Crystallite size (nm)	
**a**	**b**	**c**	**V/Z**		**Space group**
9.031 ± 0.003	9.031 ± 0.003	10.639 ± 0.002	216,921 ± 0.056	25 ± 11	P42/mnm

Supplementary Table 1. Rietveld refinement parameters of $Na_3(VO_{0.77})_2(PO_4)_2F_{1.46}$.

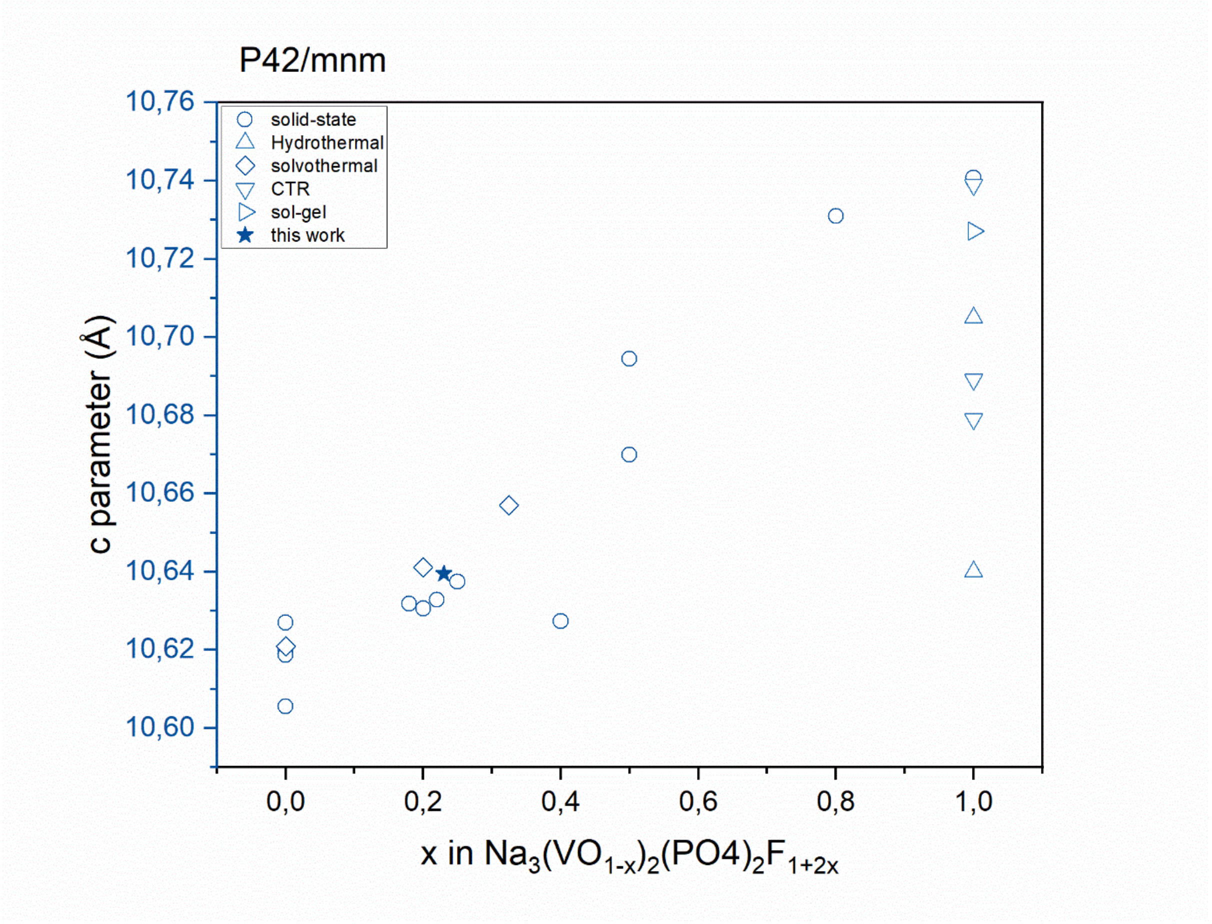

Supplementary Figure 2. The relationship between oxygen content in $Na_3(VO_{1-x})_2(PO_4)_2F_{1+2x}$ ($0 \leq x \leq 1$) and c-parameter of the unit cell.[1–14]

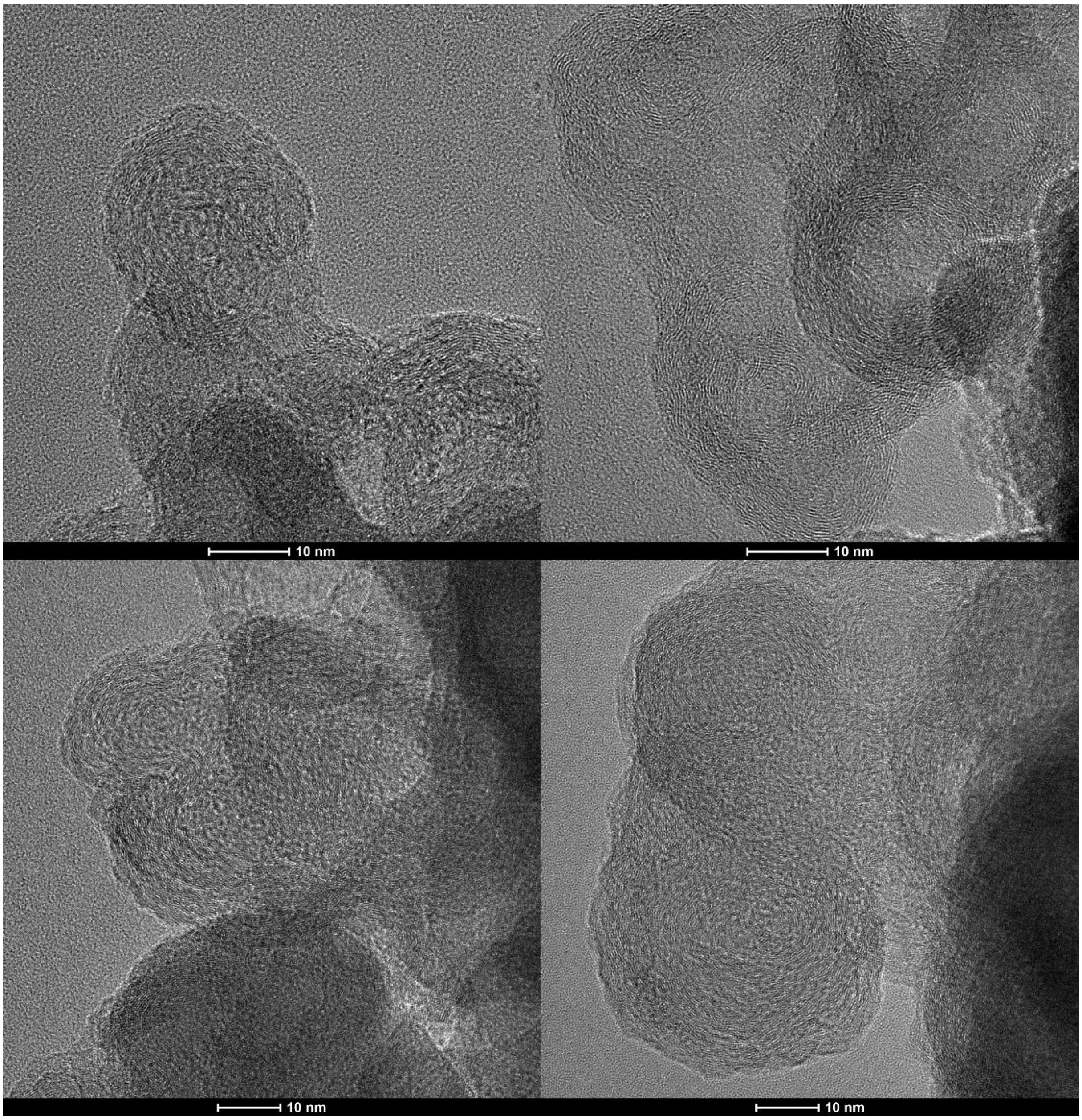

Supplementary Figure 3. Onion-like carbon structures present on the surface of NVPF@C composite powder.

Material composition*	Carbon source	Carbon content	Synthesis method	Capacity at 1 C (mAh g^{-1})	Capacity at 5 C (mAh g^{-1})	Capacity retention	NVP impurity occurrence	Ref.
$Na_3V_2(PO_4)_2F_3$	Pitch	12.4%	Solid state	99.8	96	91.9% after 500 cycles at 5 C	YES	15
$Na_3V_2(PO_4)_2F_3$	Tannic acid	6.93%	Sol-gel	112.6	102.4	90.2% after 50 cycles at 0.2 C	YES	16
$Na_3V_2(PO_4)_2F_3$	glycerol/ polyglycerol	12.44%	Microwave-assisted hydrothermal	102	74	95,3% after 200 cycles at 1 C	NO	17
$Na_3V_2(PO_4)_2F_3$	glucose	1.7%	Solid state	112.3 (initial)	unknown	89,7% after 100 cycles at 1 C	YES	18
$Na_3V_2(PO_4)_2F_3$	SWCNTs	13.54%	Hydrothermal	110.2	104.7	92,4% after 100 cycles at 0.5 C	NO	19
$Na_3V_2(PO_4)_2F_3$	Citric acid and CMC	6%	Sol-gel	106.9	93.1	74% after 500 cycles at 10 C	NO	20
$Na_3V_2(PO_4)_2F_3$	Citric acid	8.5%	Sol-gel	107.7	97.5	96.5% after 200 cycles at 1 C	NO	21
$Na_3(VO)_2(PO_4)_2F$	GO	3.5%	Open-system in-situ crystal growth	109.2	102.2	79.4% after 500 cycles at 2 C	NO	22
$Na_3(VO)_2(PO_4)_2F$	Glucose	17.5%	Hydrothermal	111	83	76.7% after 1400 cycles at 5 C	NO	23
$Na_3V_2(PO_4)_2F_3$	Citric acid	4.1%	Solid state	104.6	87.7	78% after 500 cycles at 1 C	YES	24
**$Na_3(VO_{0.77})_2(PO_4)_2F_{1.46}$**	**Vulcan XC72R**	**6.9%**	**Solution-combustion**	**110.5****	**105.2****	**94,4% after 200 cycles at 1 C**	**NO**	**THIS WORK**

Supplementary Table 2. Comparison of literature data for NVPF powders synthesised by various methods (*– claimed by the authors, **– in relation to the mass of the NVPF@C composite, 118.7 and 113.0 mAh g^{-1}, respectively in regard to the NVPF mass).

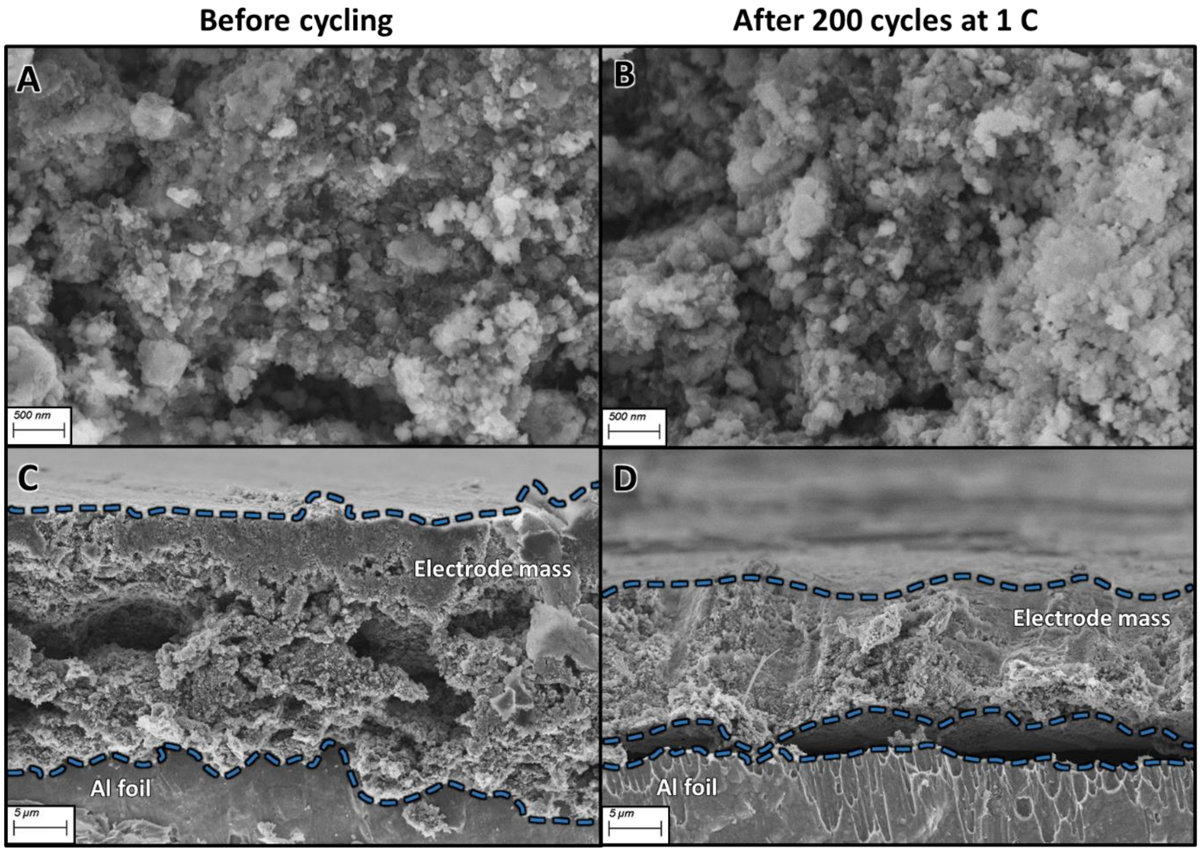

Supplementary Figure 4. SEM cross section images of NVPF@C electrode before cycling at 50 000 X (A) and 5000 X (C) magnification and electrode after 200 cycles at 1 C at 50 000 X (B) and 5000 X (D) magnification. Blue dashed lines represent edges of the aluminium foil and the electrode mass.

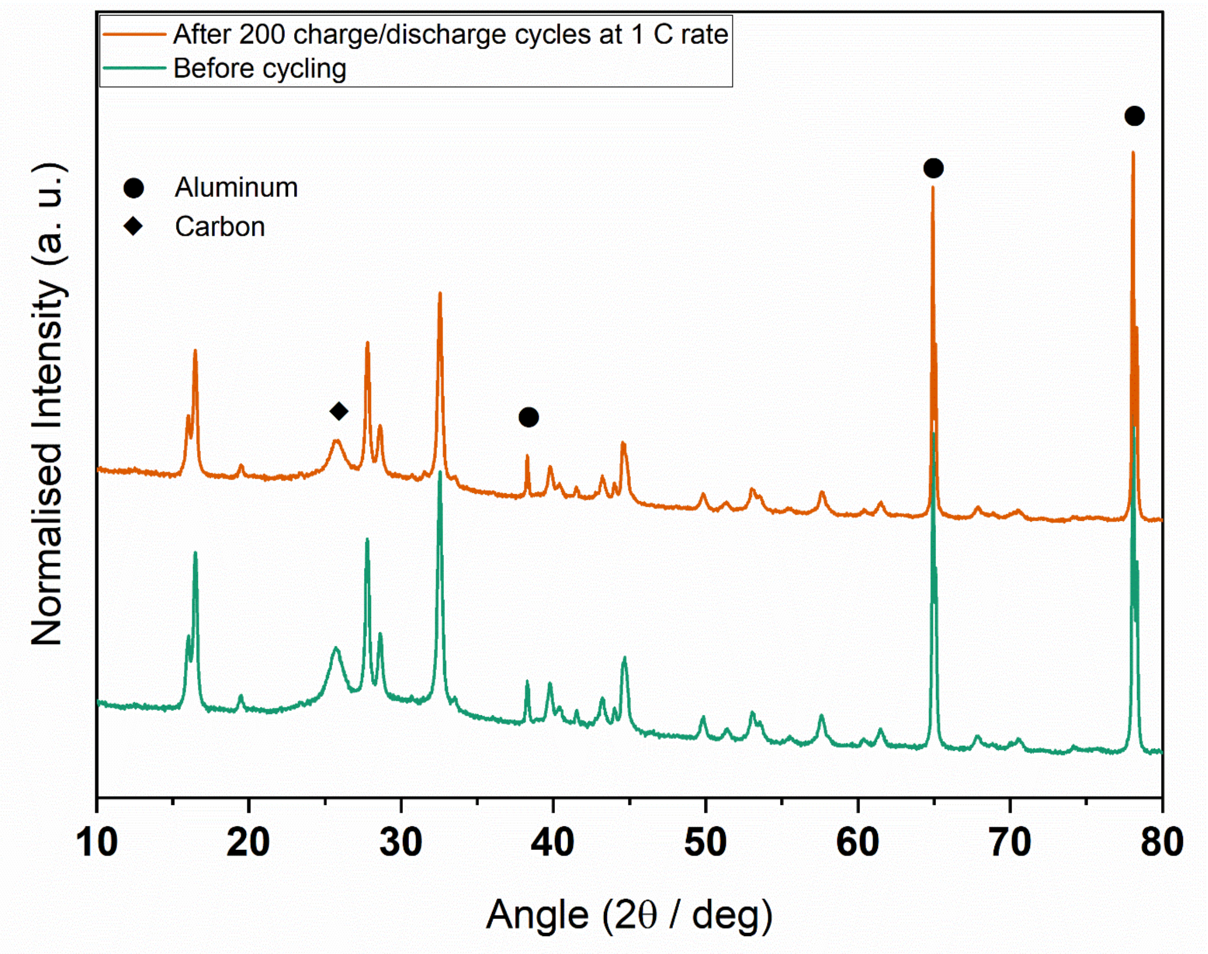

Supplementary Figure 5. XRD patterns of NVPF@C electrodes before and after 200 charge/discharge cycles at 1 C rate.

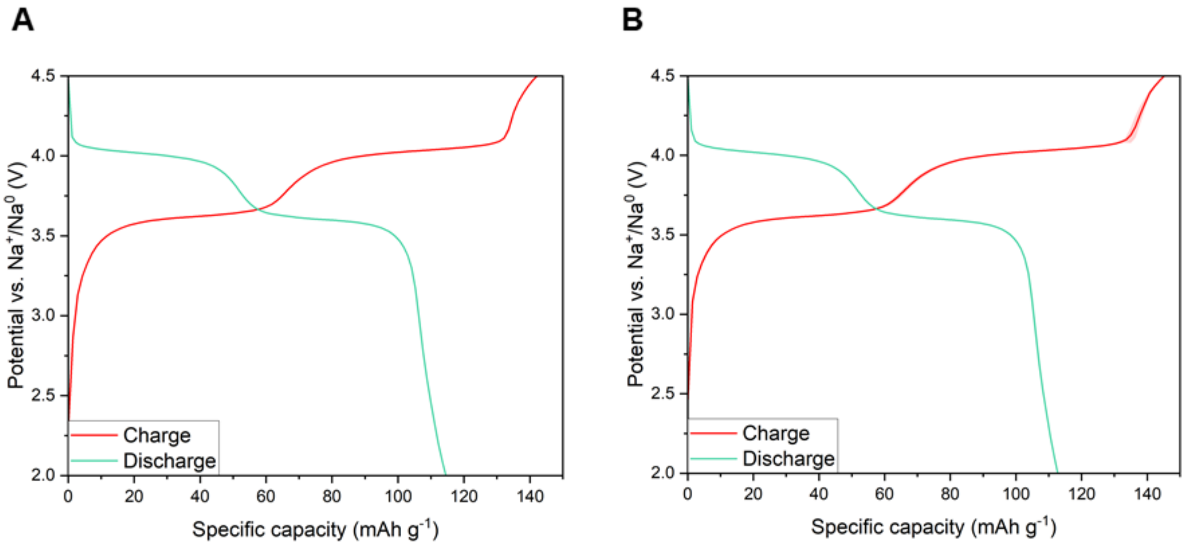

Supplementary Figure 6. Preliminary charge/discharge curves of NVPF@C composite powder during cyclability (A) and high-rate (B) tests at 0.1 C rate. Blurred areas correspond to the standard deviation of the arithmetic mean acquired from NVPF@C performance between different electrochemical cells.

v ((V s^{-1})$^{1/2}$)	$I_{pa,1}$ (A)	$I_{pa,2}$ (A)	$I_{pc,1}$ (A)	$I_{pc,2}$ (A)
0.1·10^{-3}	(1.86 ± 0.10)·10^{-4}	(2.11 ± 0.10)·10^{-4}	(1.88 ± 0.18)·10^{-4}	(2.17 ± 0.21)·10^{-4}
0.2·10^{-3}	(4.47 ± 0.26)·10^{-4}	(4.40 ± 0.31)·10^{-4}	(3.30 ± 0.74)·10^{-4}	(4.59 ± 0.22)·10^{-4}
0.5·10^{-3}	(8.00 ± 0.24)·10^{-4}	(8.75 ± 0.28)·10^{-4}	(7.21 ± 1.06)·10^{-4}	(1.07 ± 0.30)·10^{-3}
1.0·10^{-3}	(1.29 ± 0.05)·10^{-3}	(1.43 ± 0.05)·10^{-3}	(1.11 ± 0.13)·10^{-3}	(1.21 ± 0.18)·10^{-3}
2.0·10^{-3}	(1.92 ± 0.22)·10^{-3}	(2.06 ± 0.18)·10^{-3}	(1.59 ± 0.27)·10^{-3}	(1.82 ± 0.22)·10^{-3}

Supplementary Table 3. Peak currents acquired from CV analysis of NVPF@C composite powder in Na-ion cells.

v ((V s^{-1})$^{1/2}$)	$E_{pa,1}$ (V)	$E_{pa,2}$ (V)	$E_{pc,1}$ (V)	$E_{pc,2}$ (V)
0.1·10^{-3}	3.669 ± 0.013	4.069 ± 0.002	3.568 ± 0.006	3.987 ± 0.014
0.2·10^{-3}	3.701 ± 0.008	4.072 ± 0.016	3.549 ± 0.012	3.984 ± 0.023
0.5·10^{-3}	3.700 ± 0.005	4.092 ± 0.003	3.523 ± 0.012	3.942 ± 0.012
1.0·10^{-3}	3.735 ± 0.002	4.110 ± 0.006	3.515 ± 0.013	3.913 ± 0.029
2.0·10^{-3}	3.761 ± 0.017	4.144 ± 0.007	3.482 ± 0.023	3.865 ± 0.047

Supplementary Table 4. Peak potentials acquired from CV analysis of NVPF@C composite powder in Na-ion cells.

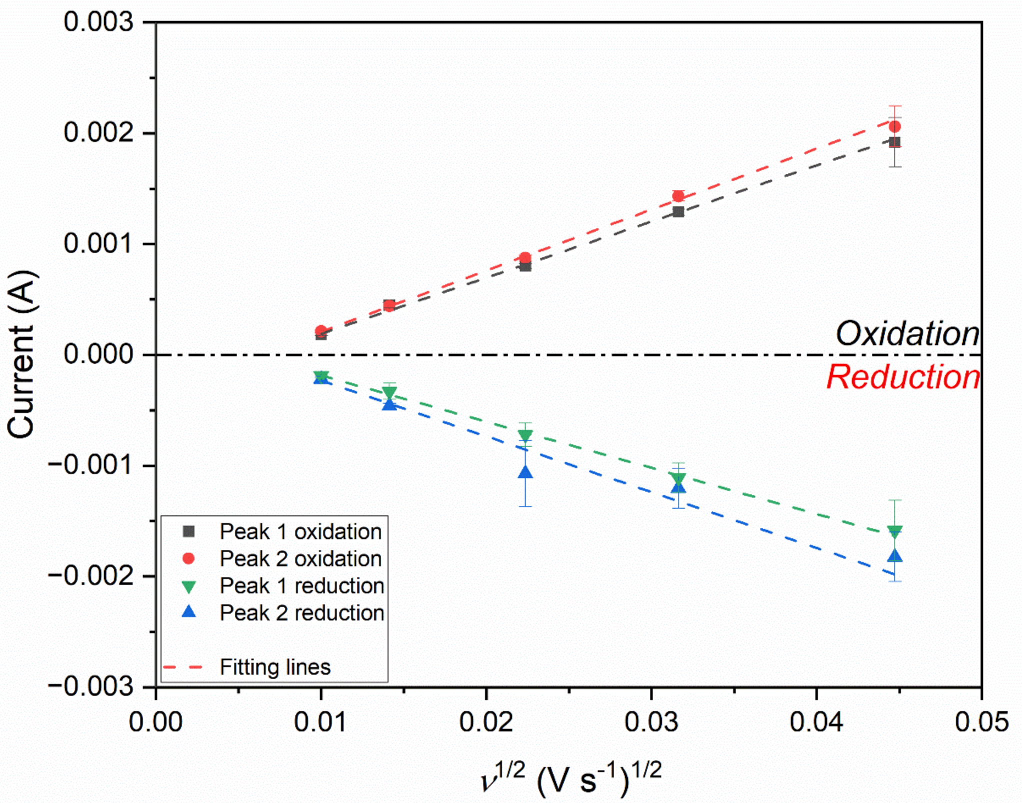

Supplementary Figure 7. Randles-Sevcik analysis of CV data acquired for NVPF@C composite powder. Error bars correspond to the standard deviation of the arithmetic mean acquired from NVPF@C performance between different electrochemical cells.

Potential vs. Na^+/Na^0 (V)	R_S (Ω)	R_{CT} (Ω)
2.0	3.30 ± 0.03	183.77 ± 1.20
4.5	3.23 ± 0.06	78.44± 1.23

Supplementary Table 5. Resistance values obtained from EIS data fitting.

References

1. Fang, R. *et al.* Impact of the F – for O 2– Substitution in Na 3 V 2 (PO 4) 2 F 3– y O y on Their Transport Properties and Electrochemical Performance. *ACS Appl. Energy Mater.* **5**, 1065–1075 (2022).

2. Chihara, K., Kitajou, A., Gocheva, I. D., Okada, S. & Yamaki, J. Cathode properties of Na3M2(PO4)2F3 [M = Ti, Fe, V] for sodium-ion batteries. *J. Power Sources* **227**, 80–85 (2013).

3. Park, Y. *et al.* A Family of High-Performance Cathode Materials for Na-ion Batteries, Na 3 (VO 1– x PO 4) 2 F 1+2 x (0 ≤ x ≤ 1): Combined First-Principles and Experimental Study. *Adv. Funct. Mater.* **24**, 4603–4614 (2014).

4. Shakoor, R. A. *et al.* A combined first principles and experimental study on Na3V2(PO4)2F3 for rechargeable Na batteries. *J. Mater. Chem.* **22**, 20535 (2012).

5. Akhtar, M., Arraghraghi, H., Kunz, S., Wang, Q. & Bianchini, M. A novel solid-state synthesis route for high voltage Na 3 V 2 (PO 4) 2 F 3−2 y O 2 y cathode materials for Na-ion batteries. *J. Mater. Chem. A* **11**, 25650–25661 (2023).

6. Le Meins, J.-M., Crosnier-Lopez, M.-P., Hemon-Ribaud, A. & Courbion, G. Phase Transitions in the Na3M2(PO4)2F3 Family (M=Al3+, V3+, Cr3+, Fe3+, Ga3+): Synthesis, Thermal, Structural, and Magnetic Studies. *J. Solid State Chem.* **148**, 260–277 (1999).

7. Song, W. *et al.* Multifunctional dual Na 3 V 2 (PO 4) 2 F 3 cathode for both lithium-ion and sodium-ion batteries. *RSC Adv.* **4**, 11375–11383 (2014).

8. Barker, J., Gover, R. K. B., Burns, P. & Bryan, A. J. Hybrid-ion: A lithium-ion cell based on a sodium insertion material. *Electrochem. Solid-State Lett.* **9**, A190 (2006).

9. Bianchini, M., Xiao, P., Wang, Y. & Ceder, G. Additional Sodium Insertion into Polyanionic Cathodes for Higher-Energy Na-Ion Batteries. *Adv. Energy Mater.* **7**, (2017).

10. Nguyen, L. H. B. *et al.* Stability in water and electrochemical properties of the Na3V2(PO4)2F3 – Na3(VO)2(PO4)2F solid solution. *Energy Storage Mater.* **20**, 324–334 (2019).

11. Yue, J. *et al.* Initiating High-Voltage Multielectron Reactions in NASICON Cathodes for Aqueous Zinc/Sodium Batteries. *Energy Mater. Adv.* **4**, (2023).

12. Sun, C., Zhang, L.-L., Deng, Z.-R., Sun, H.-B. & Yang, X.-L. Achieving High-Performance Na 3 V 2 (PO 4) 2 F 3 Cathode Material through a Bifunctional N-Doped Carbon Network. *ACS Appl. Mater. Interfaces* **16**, 35179–35189 (2024).

13. Serras, P. *et al.* High voltage cathode materials for Na-ion batteries of general formula Na3V2O2x(PO4)2F3−2x. *J. Mater. Chem.* **22**, 22301 (2012).

14. Tsirlin, A. A. *et al.* Phase separation and frustrated square lattice magnetism of Na 1.5VOPO4F0.5. *Phys. Rev. B - Condens. Matter Mater. Phys.* **84**, 014429 (2011).

15. Wang, M. *et al.* Synthesis and electrochemical performances of Na 3 V 2 (PO 4) 2 F 3 /C composites as cathode materials for sodium ion batteries. *RSC Adv.* **9**, 30628–30636 (2019).

16. Jiang, N.-B., Zhang, L.-L., Cui, C.-X., Gao, L. & Yang, X.-L. Synthesis and Electrochemical Performance of Uniform Carbon-Coated Na 3 V 2 (PO 4) 2 F 3 Using Tannic Acid as a Chelating Agent and Carbon Source. *ACS Appl. Energy Mater.* **5**, 249–256 (2022).

17. Mao, Y., Zhang, X., Zhou, Y. & Chu, W. Microwave-assisted synthesis of porous nano-sized Na3V2(PO4)2F3@C nanospheres for sodium ion batteries with enhanced stability. *Scr. Mater.* **181**, 92–96 (2020).

18. Deng, L. *et al.* Facile one-step carbothermal reduction synthesis of Na3V2(PO4)2F3/C serving as cathode for sodium ion batteries. *Electrochim. Acta* **298**, 459–467 (2019).

19. Liu, S. *et al.* Na 3 V 2 (PO 4) 2 F 3 –SWCNT: a high voltage cathode for non-aqueous and aqueous sodium-ion batteries. *J. Mater. Chem. A* **7**, 248–256 (2019).

20. Liang, K., Zhao, H., Li, J., Huang, X. & Ren, Y. High-performance Na3V2(PO4)2F3 cathode obtained by a three-in-one strategy for self-sodium compensation, interface modification, and crosslinked carbon coatings. *Appl. Surf. Sci.* **615**, 156412 (2023).

21. Qin, Y. *et al.* Effect of chelator content on the structural and electrochemical performance of Na 3 V 2 (PO 4) 2 F 3 by sol–gel preparation. *CrystEngComm* **24**, 4519–4526 (2022).

22. Yang, X., Wang, M., Xiang, X., Liu, S. & Chen, C. An open-system synthesis approach to achieve high-rate Na3(VO)2(PO4)2F/C microcubes cathode for sodium-ion batteries. *J. Electroanal. Chem.* **956**, 118088 (2024).

23. Tong, Z. *et al.* One-Step Synthesis of Carbon-Coated Na3(VOPO4)2F Using Biomass as a Reducing Agent and Their Electrochemical Properties. *Waste and Biomass Valorization* **11**, 2201–2209 (2020).

24. Zhang, J. *et al.* A surface-modified Na 3 V 2 (PO 4) 2 F 3 cathode with high rate capability and cycling stability for sodium ion batteries. *RSC Adv.* **14**, 13703–13710 (2024).
